# Modulation of cholesterol transport by maternal hypercholesterolemia in human full-term placenta

**DOI:** 10.1371/journal.pone.0171934

**Published:** 2017-02-15

**Authors:** Ran Zhang, Shan Dong, Wei-wei Ma, Xue-ping Cai, Zhi-yin Le, Rong Xiao, Qi Zhou, Huan-ling Yu

**Affiliations:** 1 School of Public Health, Beijing Key Laboratory of Environmental Toxicology, Capital Medical University, Beijing, People's Republic of China; 2 Xuanwu hospital, Capital Medical University, Beijing, People's Republic of China; Hospital Authority, CHINA

## Abstract

The significance of maternal cholesterol transporting to the fetus under normal as well as pathological circumstances is less understood. The objective of this study was to observe the effects of maternal hypercholesterolemia on placental cholesterol transportation. Human full-time placenta, maternal and venous cord blood were sampled at delivery from the pregnant women with serum total cholesterol (TC) concentrations at third trimester higher than 7.25 mM (n = 19) and the pregnant women with normal TC concentrations (n = 19). Serum lipids and expression of genes related to cholesterol transportation were measured by western blot or real-time PCR. The results indicated that serum TC, high density lipoprotein cholesterol (HDL-C), and low density lipoprotein cholesterol (LDL-C) levels were significantly increased, in pregnancies, but decreased in cord blood in hypercholesterolemic group compared to the matched control group. All the subjects were no-drinking, non-smoker, and gestational disease free. The mRNA expression of lipoprotein receptors, including LDLR and VLDLR were significantly increased, while the protein expression of PCSK9 was significantly increased in hypercholesterolemic placenta. In conclusion, maternal hypercholesterolemia might decrease the transportation of cholesterol from mother to fetus because of the high levels of PCSK9 protein expression.

## Introduction

During the normal gestation, pregnant women show an increase in serum total cholesterol (TC), low density lipoprotein cholesterol (LDL-C), and triglycerides (TG) levels from first to third trimester [1,2]. Cholesterol, the key composition of cell membranes, metabolic regulators (oxysterols) and precursor of steroid hormones in human bodies, is important for fetal development as a modulator of hedgehog signaling [3]. During the gestation [3], cholesterol in maternal-fetal transport across the placental barrier to sustain the normal fetal development though the fetus can synthesize cholesterol endogenously [4].

Maternal cholesterol was firstly taken up by lipoprotein receptors localized on the human placenta including the low-density lipoprotein receptor (LDLR), the very low density lipoprotein receptor (VLDLR), and the scavenger receptor class B type I (SRBI) [5–7]. Among them, the LDLR is responsible for binding and the internalization of LDL particles and regulating the plasma LDL level [8]. The internalized cholesterol is then either used by the placenta for synthesizing hormones or transferred to the fetus through liver X receptor (LXR) -induced up-regulation of ATP-Binding Cassette Transporter A1 (ABCA1) and G1 (ABCG1) [9].

The increase of serum cholesterol levels has been considered as a normal pregnancy physiologic change with little clinical relevance [10]. And the significance of maternal cholesterol transporting to the fetus under normal as well as pathological circumstances is also less understood. Recently, studies suggest that maternal hyperlipidemia were associated with large for gestational age (LGA) baby [11,12], preeclampsia [13], preterm birth (PTB) [14] and pregnancy-induced hypertension (PIH) [15]. Even, the gestational hypercholesterolemia also resulted in the residence of lipid on the aortas of fetuses of 6-month-old [16].

Few studies suggest that maternal contribution can vary with the maternal metabolic environment during pregnancy [9,17]. Therefore, the objectives of the present study were to (1) analyze the impact of maternal hypercholesterolemia on the expression of cholesterol metabolism genes in human term placenta and (2) to evaluate if there is any correlation between maternal/cord serum cholesterol with the expression of theses transporters.

## Materials and methods

### Subjects & recruitment

The pregnant women were recruited at the first time of prenatal visit, before their 12^th^ week of pregnancy at Bei Jing Xuanwu hospital, from Jan to Dec 2014. The length of the follow up period is about 28weeks from the first prenatal visit (before their 12th week of pregnancy) to the delivery. This study received ethics approval from the ethical committee of Capital Medical University and Xuanwu Hospital. Written informed consent was obtained from all participants before their enrollment in the study. Pregnant women with endocrine, metabolic disorder (such as diabetes, hypertension, and hypercholesterolemia), severe infectious diseases, and those with multiple pregnancies as well as premature birth, asphyxia in offspring were excluded. Pregnant women who conceived the fetus using artificial methods including in-vitro fertilization were also excluded. There were no data was available about family hypercholesterolemia diagnosis in the recruited pregnant women. After signing a consent form, each woman was interviewed and the information containing general sociodemographic data, medical history, drinking, and smoking habit was obtained by a questionnaire.

There is no definition of gestational hyperlipidemia or hypercholesterolemia and no cutoff value for normal serum cholesterol level for pregnant women. According to the reference [18], the women with serum TC concentration at third trimester (36^th^ week of pregnancy) lower than 7.25 mM were grouped to the control group (n = 19) while women with TC level higher than 7.25 mM were grouped to the hypercholesterolemia group (n = 19). All the subjects were no-drinking, non-smoker, and gestational disease free. The pregnant women in control group were matched with hypercholesterolemia group at age, pre-gestational BMI, and the fetal gender. A follow-up was made to assess body weight and height of the neonates.

### Blood and tissue samples

Fasting venous blood samples were collected at first and third trimester from the subjects. At delivery, the venous cord blood and arterial cord blood were collected in gel Vacutainer tube (BD, Oakville, ON, Canada). A portion of placentas were obtained and the amnion, the chorion and the decidual layer were removed Villous tissue was cut into approximately 5 cm^2^ pieces and kept at -80°C for further use.

The serum levels of TC, LDL-C, HDL-C, and TG were measured individually using the Unicel 36 DX600 Synchron Clinical System (Beckman-Coulter, Mississauga, ON, Canada).

### Total RNA extraction and real-time RT-PCR

Total RNA was extracted from placenta tissue using the Trizol reagent (Invitrogen) and reverse transcribed using the Revert Aid First Strand cDNA Synthesis Kit (Thermo Scientific) and Oligo-dT primers (Thermo Scientific) according to the manufacturer’s instructions.

Real-time RT-PCR was performed on the CFX Connect Real-Time Systerm (BIO-RAD) with 480 ng of cDNA template and specific primers using a Thermo Scientific Maxima SYBR Green qPCR Master Mix (Thermo Scientific) according to the manufacturer's protocols. Primers for each gene are listed in Table 1 [9,19–21] were synthesized by Sangon Biotech (Shanghai). PCR conditions were 95°C for 10 min; 59°C for 30 s, and 72°C for 30 s, 40 cycles. The 2^-ΔΔCT^ was used to calculate the relative mRNA expression levels.

**Table 1:** Primers used for real-time PCR.

Gene	Forward primer set (5’-3’)	Reverse primer set (5’-3’)
LDLR	GACGTGGCGTGAACATCTG	CTGGCAGGCAATGCTTTGG
VLDLR	TACGCTGTTGTGGAAATGTGAT	ATTCAGCACACGTCTTCTTTACA
SRBI	CGGCTCGGAGAGCGACTAC	GGGCTTATTCTCCATGATCACC
ABCA1	ACCCACCCTATGAACAACATGA	GAGTCGGGTAACGGAAACAGG
ABCG1	CAGGAAGATTAGACACTGTGG	GAAAGGGGAATGGAGAGAAGA
PPARα	AGCTTTGGCTTTACGGAATACCA	CCACAGGATAAGTCACCGAGGA
PPARγ	TCAGGGCTGCCAGTTTCG	CCCTCGGATATGAGAACCC
LXRα	AGGGCTGCAAGGGATTCTTCC	TCTGACAGCACACACTCCTCCC
LXRβ	GGAGCTGGCCATCATCTCA	GTCTCTAGCAGCATGATCTCGATAGT
PCSK9	ATGGGGCTCTGGTGGCGTGA	TCGACGTCGCTGCGGAAACC
GAPDH	GAAGGTGAAGGTCGGAGTCAA	GGAAGATGGTGATGGGATTTC

### Proteins extraction and western blot analysis

The placenta samples (200 mg) were homogenized in 2 ml of lysing buffer using a Fluka homogenizer for 10s for three times on ice. After incubation on ice for 20 min, homogenates were centrifuged at 13,000 g for 20 min at 4°C. Supernatants were collected and protein concentration was measured using a BCA protein detection kit (Biosinoble, China). We separated the proteins on 12% SDS polyacrylamide gels under reducing conditions and transferred them onto PVDF membranes (Bio-Rad, USA). Subsequently, membranes were blocked in 10% bovine serum albumin in Tris buffered saline with 0.1% Tween 20 for 30 minutes. Membranes were incubated with primary antibody (PCSK9 antibody, 1:500 and β-actin antibody, 1:5000) at 4°C for overnight. After five washings with Tris buffered saline containing 0.1% Tween 20 for 3 minutes, membranes were incubated with second antibody at a 1:10000 dilution for 40 minutes. After washing, the membranes were developed with ECL western blotting reagents according to the manufacturer’s instructions. Films were scanned and quantified by using Image-Quant software (Molecular Dynamics).

### Statistical analyses

Data were expressed as the means ± SD or SEM, and analyzed with the paired Student’s t-test at p < 0.05 level of significance, to evaluated difference between groups. For the relationship between two variables of the same population, results are expressed as Pearson’s correlation and the curve represent Pearson’s linear correlation. All statistical analyses were performed using the SPSS software 13.0.

## Results

### Population characteristics

Characteristics of pregnancies and neonates were presented in Table 2. Both in control and hypercholesterolemia group, the age of women was 29 years old. The gestational age was significant longer in hypercholesterolemia women. No significant difference was observed in the pre-gestational BMI, bodyweight gain, newborn birth weight and height, blood pressure, and blood glucose.

**Table 2:** Population characteristics.

	Control (n = 19)	Hypercholesterolemia (n = 19)	P value
age (year)	29.53±3.08	29.05±2.86	0.626
Gestational age (week)	39.03±0.92	39.73±1.12	0.040
Pre-gestational BMI (kg/m^2^)	22.28±3.04	21.82±3.03	0.644
Body weight (kg)			
Pre-gestation	58.22±9.05	57.57±8.83	0.825
Second trimester	68.16±9.73	68.37±9.62	0.947
Third trimester	72.90±9.08	73.25±10.07	0.909
Body weight gain (kg)	13.68±3.54	15.05±6.40	0.410
Newborn birth weight (g)	3466±466	3468±392	0.985
Newborn height (cm)	50.18±1.47	50.22±1.72	0.955
Diastolic blood pressure (mm Hg)			
First trimester	75.22±9.93	71.94±9.24	0.155
Second trimester	68.89±8.14	66.18±7.40	0.311
Third trimester	71.50±8.43	74.21±6.72	0.286
Systolic Blood Pressure(mm Hg)			
First trimester	111.39±12.98	105.65±10.08	0.319
Second trimester	110.56±9.38	107.65±10.77	0.400
Third trimester	112.50±10.88	114.74±11.36	0.545
Fasting blood glucose (g/L)	4.27±0.34	4.25±0.35	0.850
OGT1h	7.27±1.33	7.23±1.52	0.931
OGT2h	5.93±1.03	6.08±1.23	0.682

Results are expressed as means ± SD where compared to control group.

### Influence of maternal hypercholesterolemia on maternal and cord blood lipids profile

In control and hypercholesterolemia women, the concentrations of serum TC, HDL-C, LDL-C, and TG were given in Table 3. At first trimester, the maternal serum TC and LDL-C concentrations were significantly higher than that in hypercholesterolemia women. At third trimester, TC, HDL-C, and LDL-C levels were significantly increased in hypercholesterolemia group compared to the control group. In all pregnant women, the serum lipids in third trimester were significantly higher than that in first trimester.

**Table 3:** Serum lipids profile in dams at fist and third trimester.

	First trimester		Third trimester	
	Control group (n = 19)	Hypercholesterlemia (n = 19)	Control group (n = 19)	Hypercholesterlemia (n = 19)
TC (mM)	4.25±0.79	4.80±0.49*	5.25±0.90^#^	7.62±0.54*^#^
TG (mM)	1.41±0.62	1.24±0.31	3.36±1.42^#^	3.38±0.98^#^
HDL-C (mM)	1.82±0.38	2.22±0.31*	1.97±0.37^#^	2.53±0.42*^#^
LDL-C (mM)	2.19±0.55	2.38±0.33	2.73±0.81^#^	4.55±0.76*^#^
HDL/LDL	0.88±0.25	0.92±0.18	0.78±0.24	0.57±0.15*^#^

* comparing to the control group, P<0.05;

^#^ comparing to the first trimester, P<0.05.

**Fig 1:**
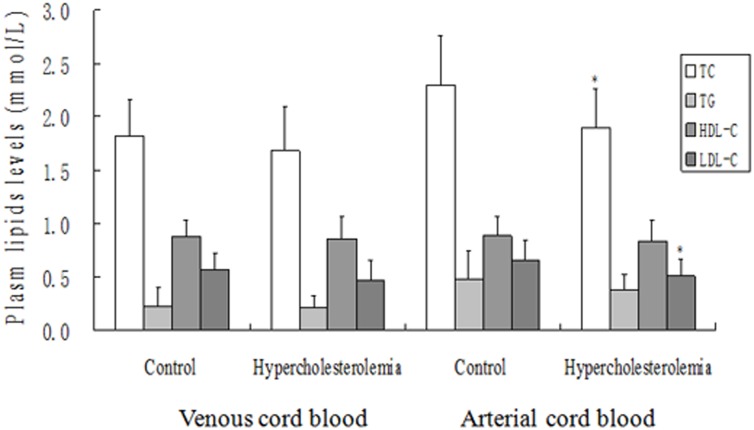
Influence of maternal hypercholesterolemia on cord blood lipid levels. * means comparing with control group P<0.05.

The plasma TC, HDL-C, LDL-C, and TG concentrations in venous cord blood were not significantly different between the two groups, while the TC and LDL-C levels in arterial cord blood were significantly lowed in hypercholesterolemia women (Fig 1).

**Fig 2:**
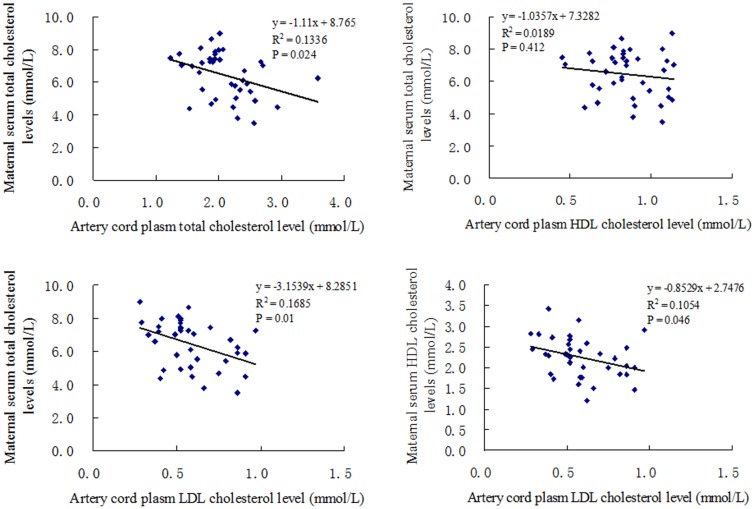
Correlation analysis between maternal serum cholesterol (third trimester) concentration and artery cord blood cholesterol concentration. Results are expressed as Pearson’s correlation and the curve represent Pearson’s linear correlation.

When the correlations between maternal serum cholesterol concentration and cord blood serum lipids were analyzed, no significant correlation were found between maternal serum TC, HDL-C, LDL-C, and TG levels at first trimester and venous cord blood lipids levels. Maternal serum TC and HDL-C concentrations at third trimester were negatively correlated with artery cord plasma LDL-C level significantly (Fig 2). Artery cord plasma TC level was found negatively to correlate with maternal serum TC levels at third trimester significantly (Fig 2).

### Influence of maternal hypercholesterolemia on mRNA expression of lipoprotein receptors, nuclear receptors, and transporters in human term placentas

**Fig 3:**
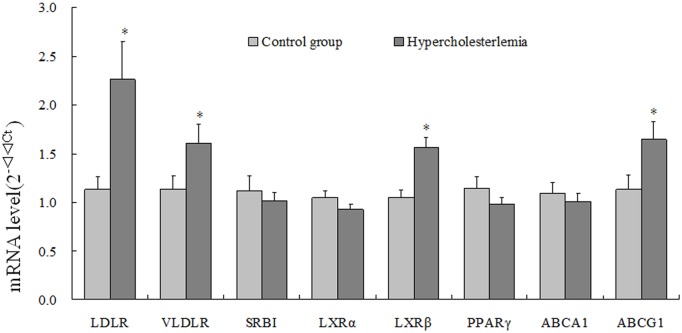
Expressions of genes related to cholesterol transporting in placenta. Real-time PCR analysis of ABCG1, LXRβ, LXRα, SRBI, PPARγ, VLDLR, ABCA1 and LDLR genes expression from placenta of control and hypercholesterolemia groups. Results are the means ± SE (n = 19).

The mRNA expression of lipoprotein receptors, including LDLR and VLDLR were significantly increased in placenta from hypercholesterolemic women comparing to control group, while the expression of SRBI was not affected significantly. Among the three nuclear receptors regulating cholesterol metabolism in cells, only the LXRβ’s expression was significantly increased. The mRNA expression of ABCG1, the membrane transporter that mediates cellular efflux of cholesterol, was significantly induced by maternal hypercholesterolemia. The mRNA expressions of other genes measured in the present study were not significant difference between in two groups (Fig 3).

### Influence of maternal hypercholesterolemia on mRNA and protein expression of PCSK9 in human term placentas

**Fig 4:**
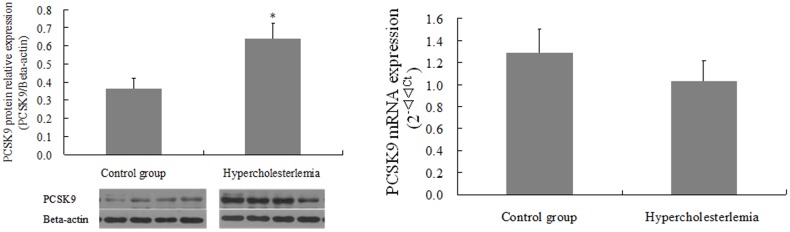
Pcsk9 protein expression (western blot, A) and mRNA expression (real-time PCR, B) in placenta from control and hypercholesterolemic pregnancy. Results are the means ± SEM (n = 17). *p < 0.01 compared to control group.

The protein expression of PCSK9 was significantly increased by maternal hypercholesterolemia compared to control group with no significantly change of mRNA expression in human term placenta (Fig 4).

## Discussion

In the present study, the lipids profile of pregnant women at first and third trimester were analyzed and the results showed that TC, TG, HDL-C, and LDL-C levels were significant higher in third trimester. The results indicated that the transportation of cholesterol to fetus was lower in hypercholesterolemic pregnancy evidenced by the significant lower artery plasma TC and LDL-C levels and significant higher expression of PCSK9 in placenta.

In fetal development, cholesterol plays an important role as it is a fundamental component of cell membranes and a precursor substance for steroid hormones [22]. The fetus can synthesize cholesterol endogenously [4], nevertheless the placenta also transports cholesterol from maternal circulation to the fetus by cholesterol-carrying lipoproteins, such as LDL, HDL and VLDL [23]. Maternal cholesterol has been shown to across the placenta and enter the fetal circulation, contributing substantially to the fetal cholesterol pool in animals [24] and humans [25]. Maternal hypercholesterolemia may pose a significant risk [26] to the fetus on account of the increase in maternal cholesterol transfer to fetus and then the formation of fatty streak in the artery [16]. So, it was supposed that cholesterol transportation increased as the maternal serum cholesterol concentration increased. Using ^3^H labeled cholesteryl oleate [27], Burke and the colleagues found that increasing maternal lipoprotein-cholesterol concentrations could enhance the cholesterol in maternal-fetal transport. It was even showed that there was a direct correlation between the concentration of maternal cholesterol and the presence of fatty streaks in fetus [16]. Vuorio[28] also showed a significant increase of cholesterol and LDL-C in the cord blood of familiar hypercholesterolemia(FH) newborns if compared with non-FH newborns. In our study, the cord blood cholesterol concentrations were lower in hypercholesterolemia group than that in control ones. And the negative correlations were found between the maternal serum cholesterol concentrations at third trimester and artery blood cholesterol levels, indicating the possibility that there were less cholesterol transportation from mothers to fetus in hypercholesterolemic pregnant women. We supposed the transfer of the cholesterol to the fetal circulation may be altered in hypercholesterolemic group. Some authors [29] have reported that there were no associations between maternal and newborn cholesterol levels. Marseille-Tremblay [30] and Ethier-Chiasson M [31] found a decrease serum TC and LDL-C concentrations in venous cord blood. So, the study design might be the major factors resulted in the discrepancy.

Placenta is a vital organ for cholesterol transfer from the maternal to the fetus. ABCG1, LXRβ, LXRα, SRBI, PPARγ, VLDLR, ABCA1 and LDLR are important factors responsible for cholesterol transportation localized on placenta [23]. In our study, we found that the mRNA expression of ABCA1 which priority expressed on the maternal side to induce a decreased cholesterol efflux [32] was unchanged, whereas the mRNA expression of ABCG1 which priority expressed on the fetal side of the placenta to provoke a decreased cholesterol efflux was significant increased in hypercholesterolemic pregnancies. Evemie Dube [9] and Anger [33] have shown that the expression of ABCA1 and ABCG1 mRNA and other ABC transporters are not changed in GDM pregnancies. The increased expression of ABCG1 could be part of a compensatory mechanism to satisfy the demand of cholesterol to the fetal circulation. A study [34] using mice found that the lacking of PCSK9 increased the protein expression of LDLR in the liver reducing the cholesterol in the circulation. The altered mRNA expression of LDLR and VLDLR could be attributed to the maternal inflammatory status [35]. There is a now growing evidence [36] that during the first weeks of life, the fetus largely depend on maternal cholesterol as its cholesterol source when most organs developed. Petar [37] found that PCSK9 mRNA is most ample in yolk sac and fetal liver. While the abundant PCSK9 degraded the cholesterol receptors to transfer the cholesterol from maternal to the fetal to support the organs develop during the early pregnancy. At the third trimester, fetus’s cholesterol is synthesized by itself, mainly by the fetal liver [38]. In this period, placental may plays a protection role in the cholesterol circulation from maternal to the fetal to prevent so much cholesterol transport to the fetal. So we found the increased level of PCSK9 protein expression may plays such a part in the circulation of cholesterol to the fetal. Leiva [39] suggested that newborn umbilical blood lipoprotein and triglyceride concentrations compared with those in maternal circulation were lower, without comparing the different between newborns.

This study has some advantages from other studies. First, the control pregnant women were matched with the hypercholesterolemic pregnant women in age, pre-gestational BMI, and fetal gender, and then, the confounders were adjusted. Second, the fasting samples were used during the first and third time of pregnancy. Some researchers measured the lipids in umbilical venous blood, but we measured both the venous and artery blood. But we also have limitations, such as the sample size is small and the DNA test for the pregnancies and the newborns is lack.

In conclusion, our study found that lipid profiles in maternal and cord blood are altered in the case of hypercholesterolemic pregnancies and the consequence of the modulation of placental LDLR and PCSK9 expression in the control of cholesterol concentrations in both term placenta and fetus. Our experiment using human placenta and thus cannot be observed the maternal cholesterol levels how inhibited the cholesterol transports from mother to fetus in full-term. Further studies would be assessed the exact influence of these changes on placental cholesterol transport with dynamic observation.

## Supporting information

S1 Fig Western blot of Pcsk9 protein expression in placenta from control and hypercholesterolemic pregnancy. (PPT)
