# Supplementary material for: Modulation of cholesterol transport by maternal hypercholesterolemia in human full-term placenta
Source: PLoS One. 2017 Feb 15;12(2):e0171934. doi: 10.1371/journal.pone.0171934 (PMC5310867; doi:10.1371/journal.pone.0171934)
Supplement: S1 Fig — (PPT) [file pone.0171934.s001.ppt]

## Slide 1
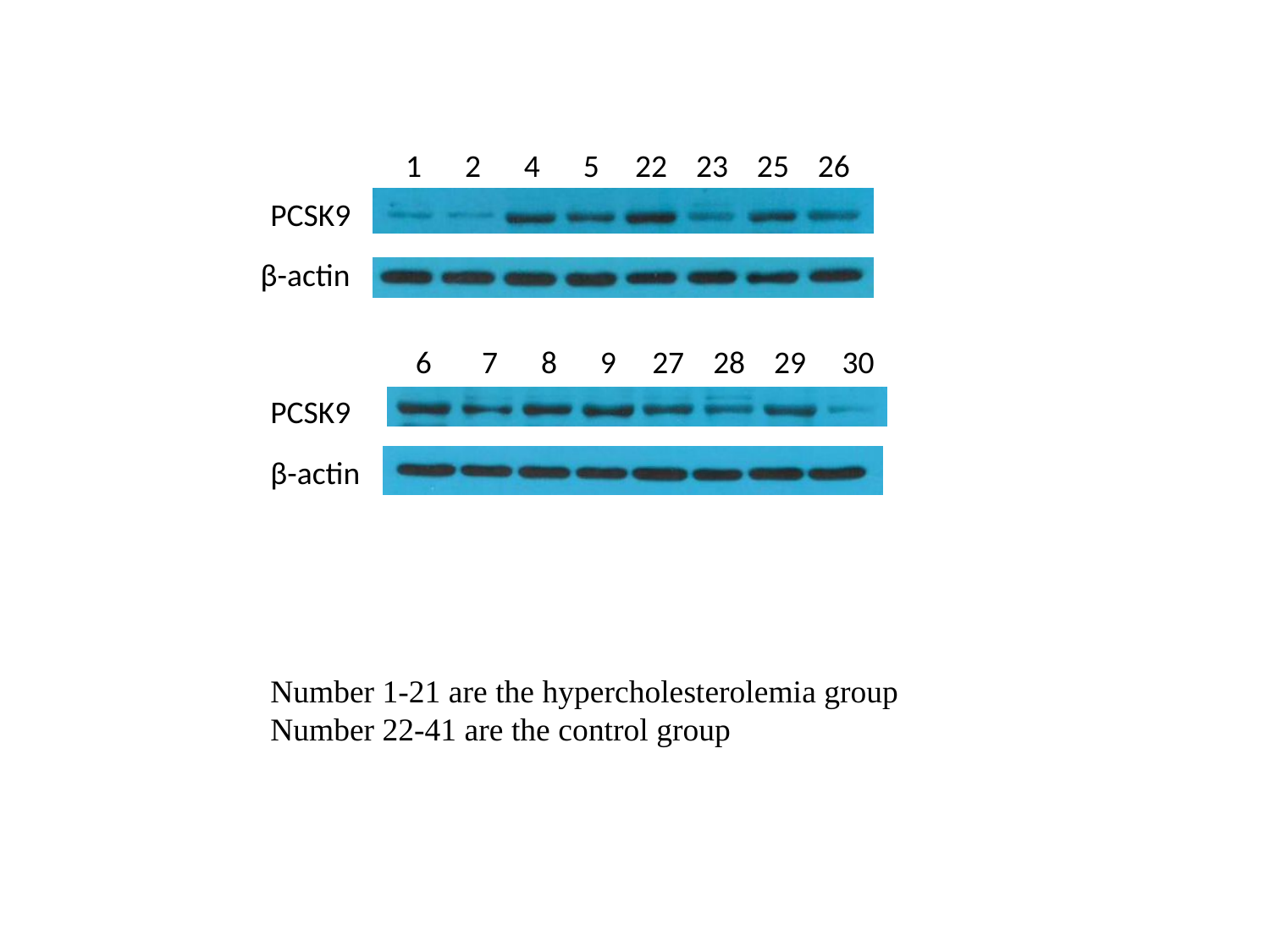

1 2 4 5 22 23 25 26
PCSK9
β-actin
 6 7 8 9 27 28 29 30
PCSK9
β-actin
Number 1-21 are the hypercholesterolemia group
Number 22-41 are the control group

## Slide 2
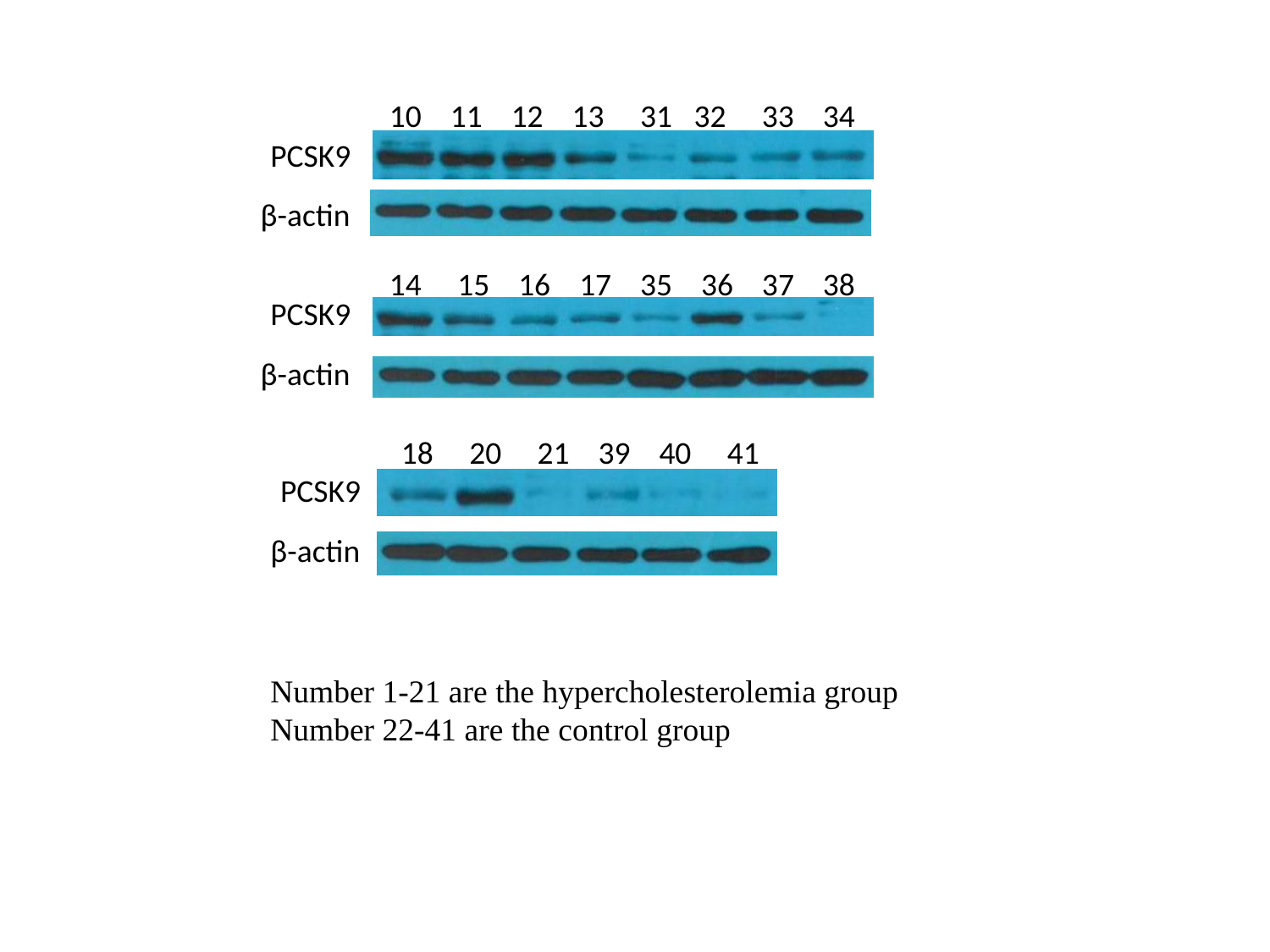

10 11 12 13 31 32 33 34
PCSK9
β-actin
14 15 16 17 35 36 37 38
PCSK9
β-actin
 18 20 21 39 40 41
PCSK9
β-actin
Number 1-21 are the hypercholesterolemia group
Number 22-41 are the control group
